# Test your knowledge and understanding

**Published:** 2013

This page is designed to test your understanding of the concepts covered in this issue and to give you an opportunity to reflect on what you have learnt. The multiple true/false questions were produced in collaboration with the International Council of Ophthalmology (ICO) and the Diagnose This quiz is provided courtesy of the Ophthalmic News and Education (ONE®) Network of the American Academy of Ophthalmology.

**Table:** 

**1.**	**Think about undernutrition and vitamin A deficiency**	**True**	**False**
**a**	Both disease and infection contribute to undernutrition and stunting.	□	□
b	The children who actually show the eye signs of vitamin A deficiency should be our main concern.	□	□
c	Children with vitamin A deficiency may go blind, but are not at increased risk of death.	□	□
d	Even if a family has enough vitamin A-rich foods, children may still be deficient.	□	□
**2.**	**Think about the sources of vitamin A**	**True**	**False**
**a**	Meat and liver are both good animal sources of vitamin A.	□	□
**b**	Sunlight can destroy vitamin A.	□	□
**c**	For children younger than 12 months, breast milk alone provides enough vitamin A.	□	□
**d**	Adding fat to the diet aids absorption of vitamin A.	□	□
**3.**	**Think about the eye signs of vitamin A deficiency**	**True**	**False**
**a**	Children usually develop night blindness first and only later develop corneal ulcers.	□	□
**b**	Children with Bitot's spots are not necessarily vitamin A deficient.	□	□
**c**	The eye signs of vitamin A deficiency are usually bilateral (in both eyes).	□	□
**d**	Children with night blindness tend to become more active at night.	□	□

## ANSWERS

**1.a. True. b. False**. Vitamin A deficiency usually affects whole communities, not just individuals. If some children have the eye signs, many more have vitamin A deficiency. **c. False**. There is a very strong link between vitamin A deficiency and death. **d. True**. Customs and local beliefs might prevent parents from giving children the right foods, particularly if they are ill.
**2.a. False**. Liver is a good source, but meat (the muscle) is not a good source. **b. True. c. False**. From 6 months, children need both breast milk and vitamin A-rich foods. **d. True**.
**3.a. False**. A child who is vitamin A deficient, but who does not have any of the eye signs, may develop corneal ulcers when infection or diarrhoea depletes the liver stores of vitamin A, causing acute deficiency. **b. True. c. True. d. False**. Mothers describe their children as becoming less active at night.

